# Retraction: Protein kinase C is a calcium sensor for presynaptic short-term plasticity

**DOI:** 10.7554/eLife.35974

**Published:** 2018-03-07

**Authors:** Diasynou Fioravante, YunXiang Chu, Arthur PH de Jong, Michael Leitges, Pascal S Kaeser, Wade G Regehr

Fioravante D, Chu YX, de Jong APH, Leitges M, Kaeser PS, Regehr WG. 2014. Protein kinase C is a calcium sensor for presynaptic short-term plasticity. *eLife* **3**:e03011. doi: 10.7554/eLife.03011.

Published 5, August 2014

The authors are retracting the *eLife* paper cited above based on irregularities associated with the electrophysiology experiments performed by YXC that were discovered by the corresponding author (WGR). The most important experiments are those in which the PKCβ is virally expressed in presynaptic cells in PKCαβ dko animals, in which the calcium sensitive PKC isoforms are eliminated in presynaptic cells and post-tetanic potentiation (PTP) is very small. In some cases the calcium sensitive wildtype, PKCβ^WT^, is expressed and PTP is rescued (Figure 1F), and in other cases mutated calcium-insensitive PKCβ^D/A^ is expressed and PTP is not rescued (Figure 3B).

A careful re-examination of these experiments revealed that for many cells in PKCβ^WT^ rescue experiments, synaptic enhancement was accompanied by an increase in the stimulus artifact and a speeding of excitatory postsynaptic current (EPSC) decays. Both of these effects were transient and had approximately the same time course as the enhancement of synaptic amplitude. The alterations in artifact and time course were not present in experiments in which PKCβ^D/A^ was expressed. No biological mechanism has been identified that can account for these changes in stimulus artifact and EPSC decay. For each PTP trial, data were collected continuously for approximately two minutes, and there was no independent measure of series resistance and series resistance compensation throughout the trial. It was assumed that changes in series resistance would be small, and that series resistance compensation would remain constant throughout each trial. These challenging experiments will need to be repeated in a verifiably blind manner.

The authors will post an update if further information becomes available in the future, but in light of the re-analysis the key conclusions of this work are now in doubt and the authors agree that the paper should be retracted.

